# Supplementary figures and images for: Activation of the β-adrenergic receptor exacerbates lipopolysaccharide-induced wasting of skeletal muscle cells by increasing interleukin-6 production
Source: PLoS One. 2021 May 18;16(5):e0251921. doi: 10.1371/journal.pone.0251921 (PMC8130926; doi:10.1371/journal.pone.0251921)

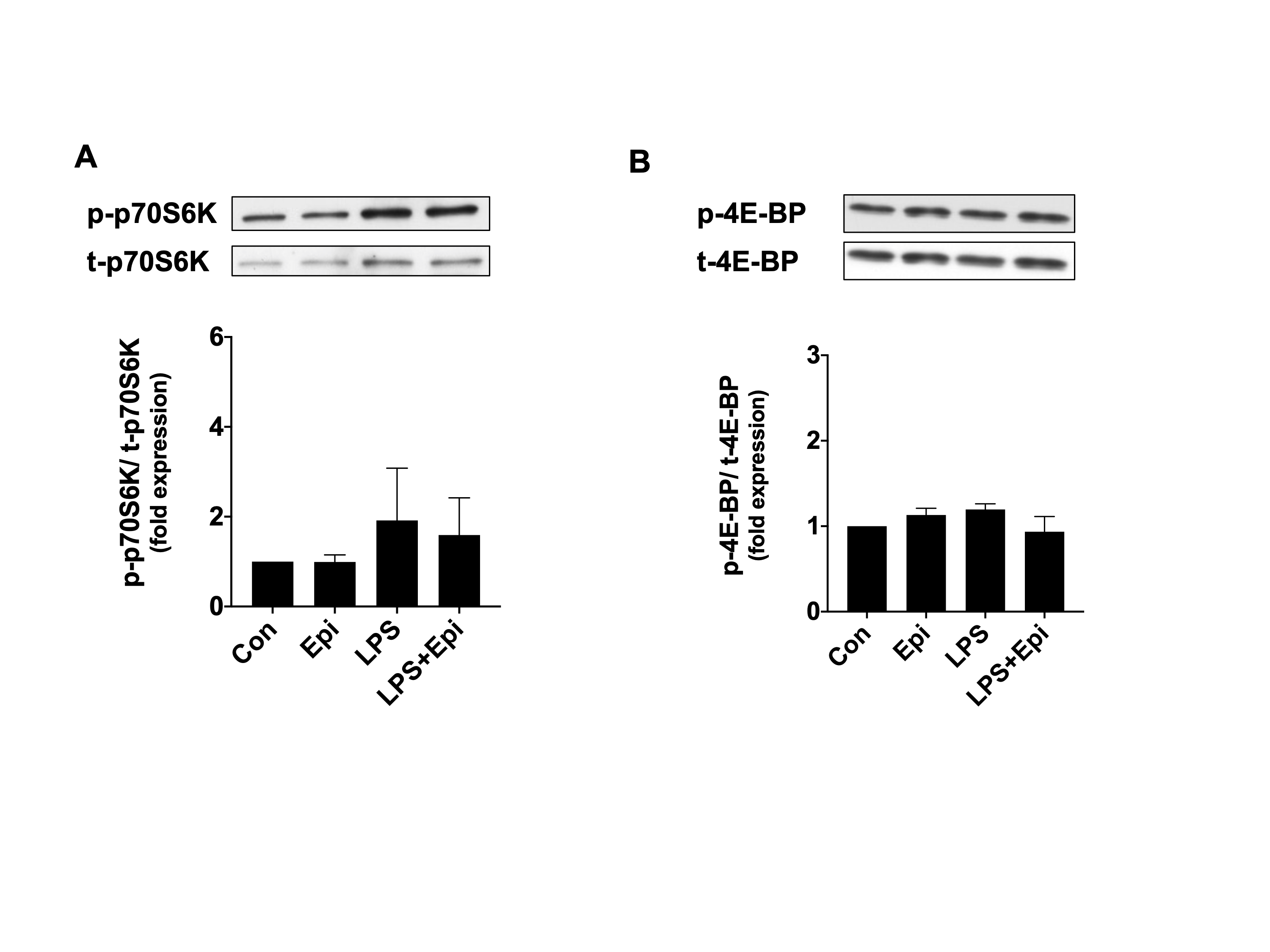

Supplement: S1 Fig — C2C12 myotubes were exposed to Epi (1 μM) and/or LPS (50 ng/ml) for 3 h. Whole-cell lysates were analyzed for p-p70S6K, t-p70S6K, p-4EBP, and t-4EBP protein expressions with immunoblotting analysis. Representative immunoblots are shown. Expressions of p-p70S6K (A) and p-4EBP (B) proteins were quantified by densitometric analysis, and were normalized to the expression level of t-p70S6K and t-4EBP, respectively. Fold induction relative to the value of the control condition is presented as mean ± SEM (n = 3). (†P < 0.05 and ††P < 0.005 compared with control, *P < 0.05 and **P < 0.005 for comparisons between the indicated groups) (TIFF) [file pone.0251921.s001.tiff]
