# Supplementary material for: Activation of the β-adrenergic receptor exacerbates lipopolysaccharide-induced wasting of skeletal muscle cells by increasing interleukin-6 production
Source: PLoS One. 2021 May 18;16(5):e0251921. doi: 10.1371/journal.pone.0251921 (PMC8130926; doi:10.1371/journal.pone.0251921)

Blot images of Fig 1A

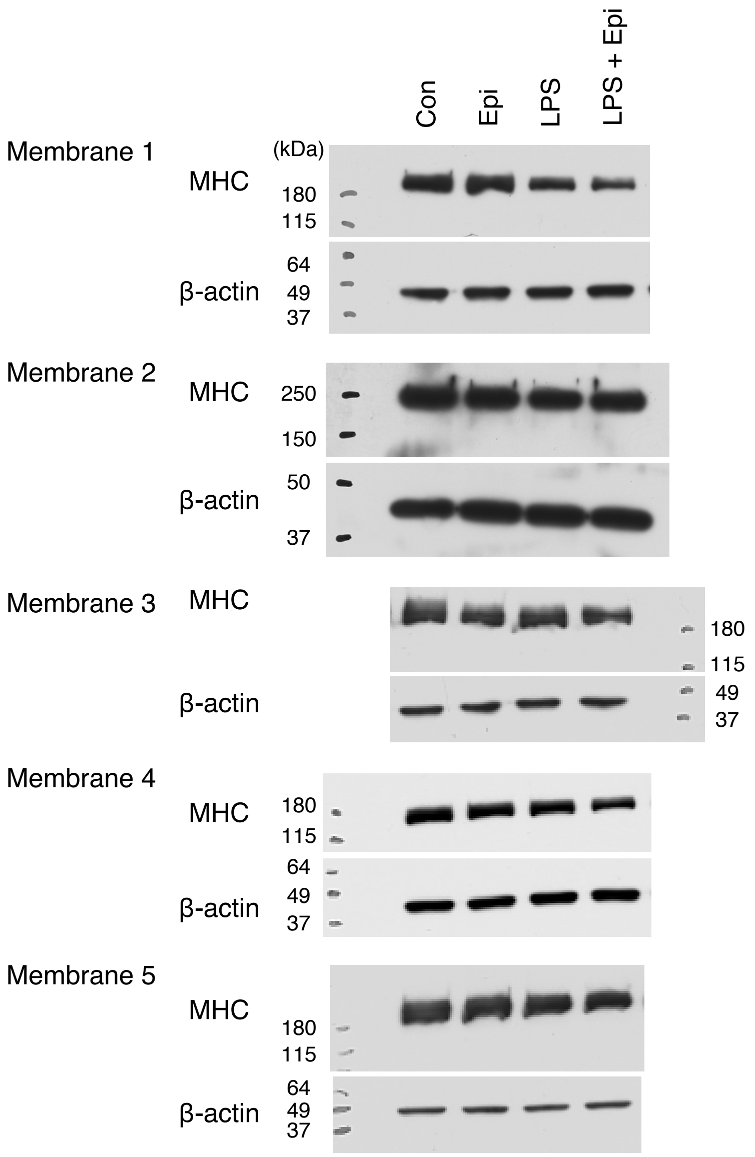

Blot images of Fig 3A

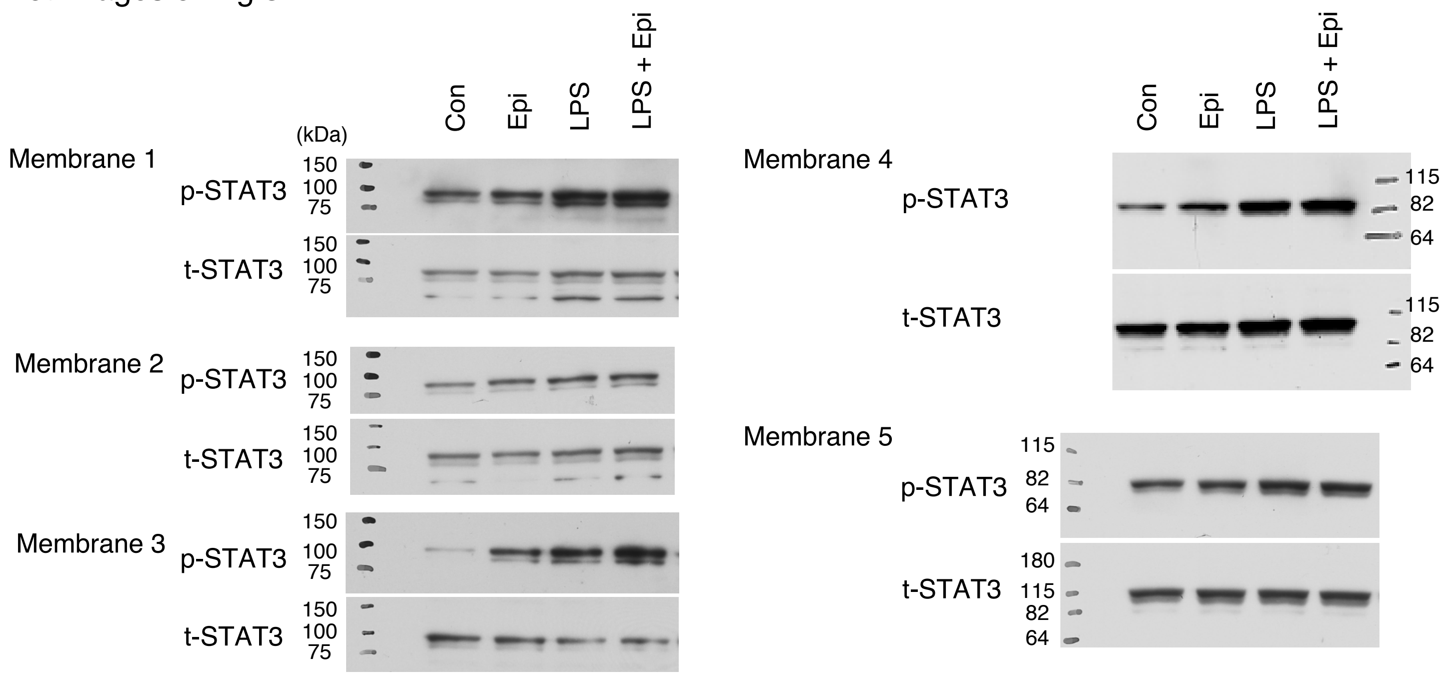

Blot images of Fig 3B

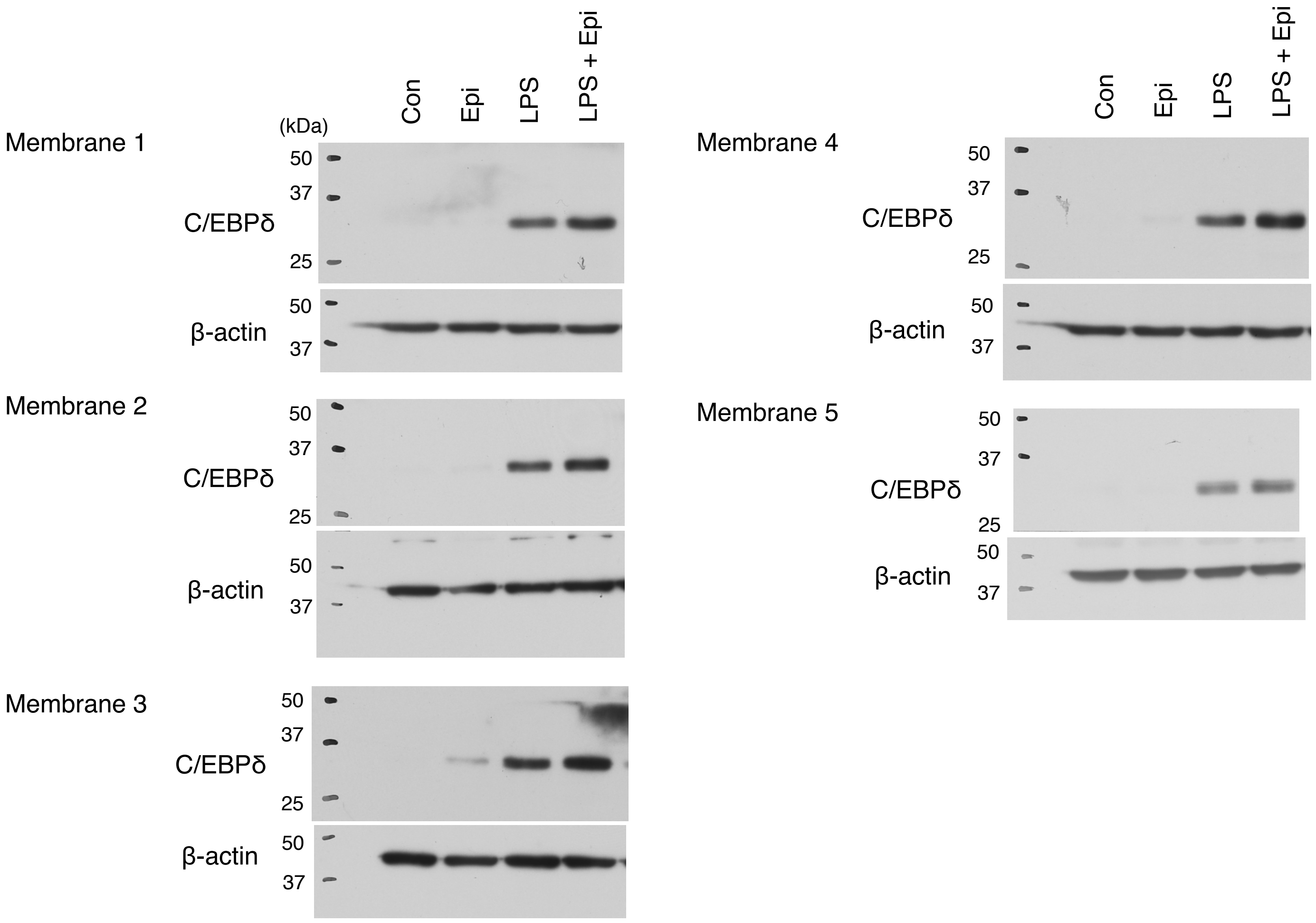

Blot images of Fig 5

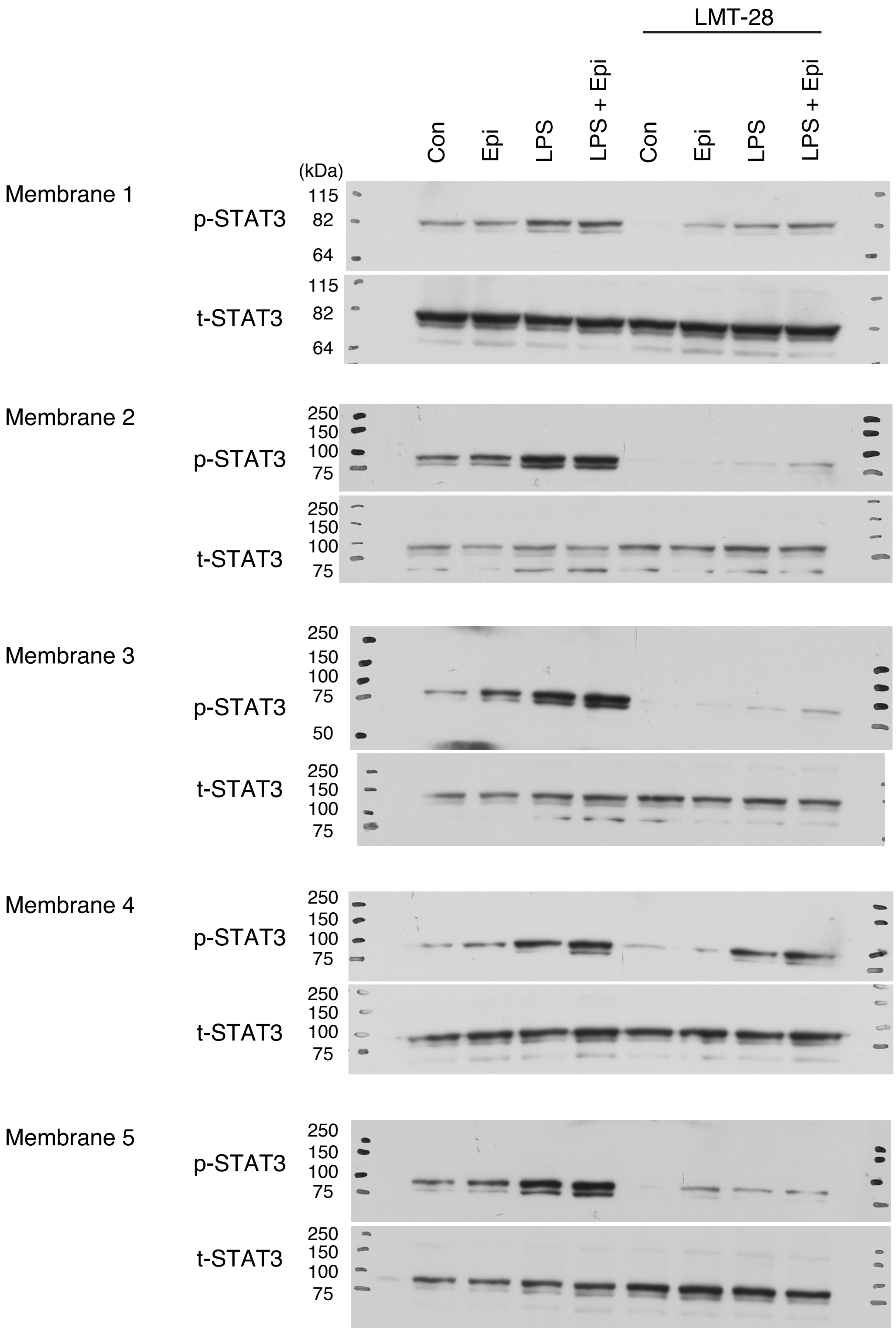

Blot images of Fig 5

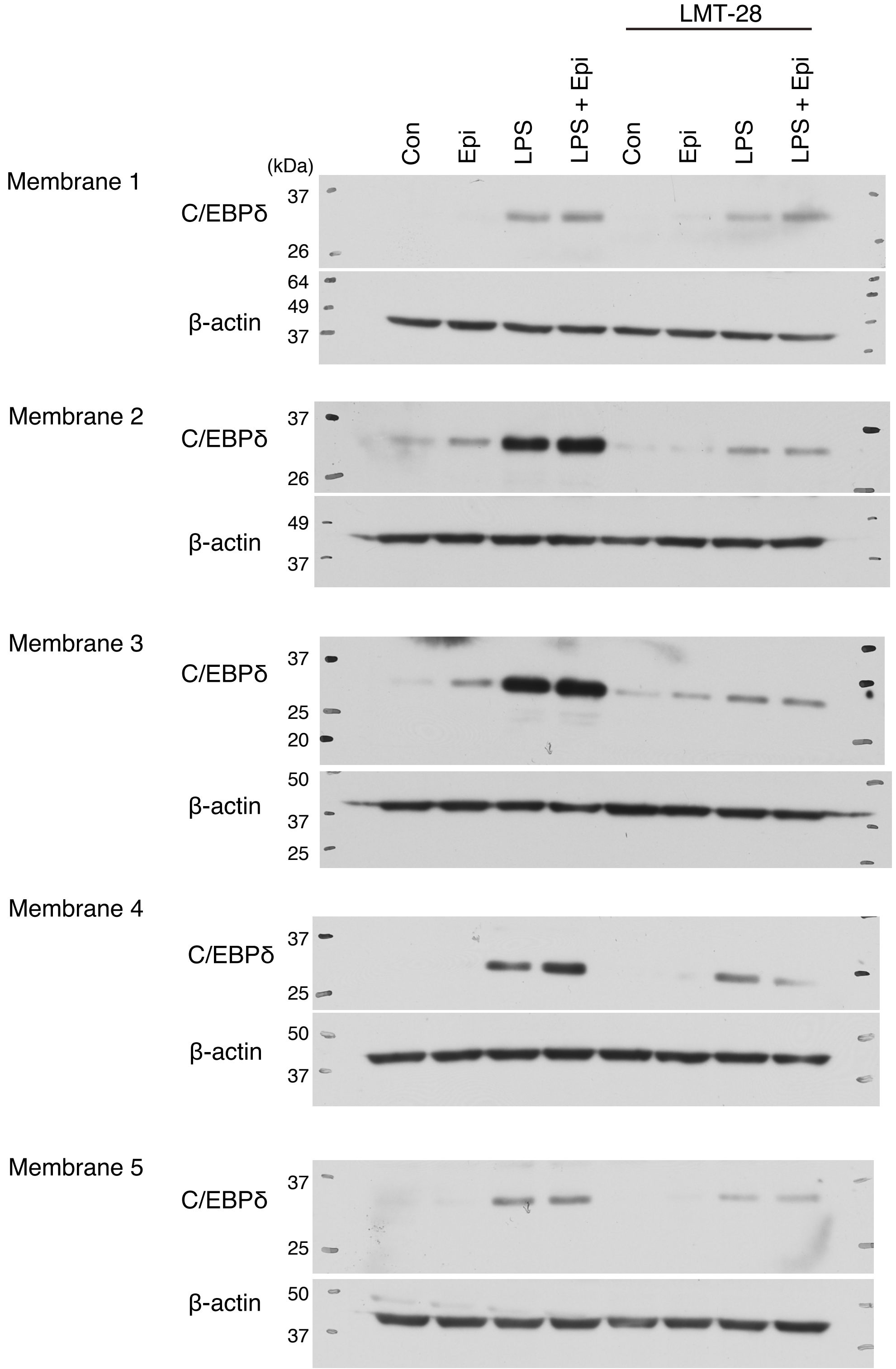

Blot images of Fig 6A

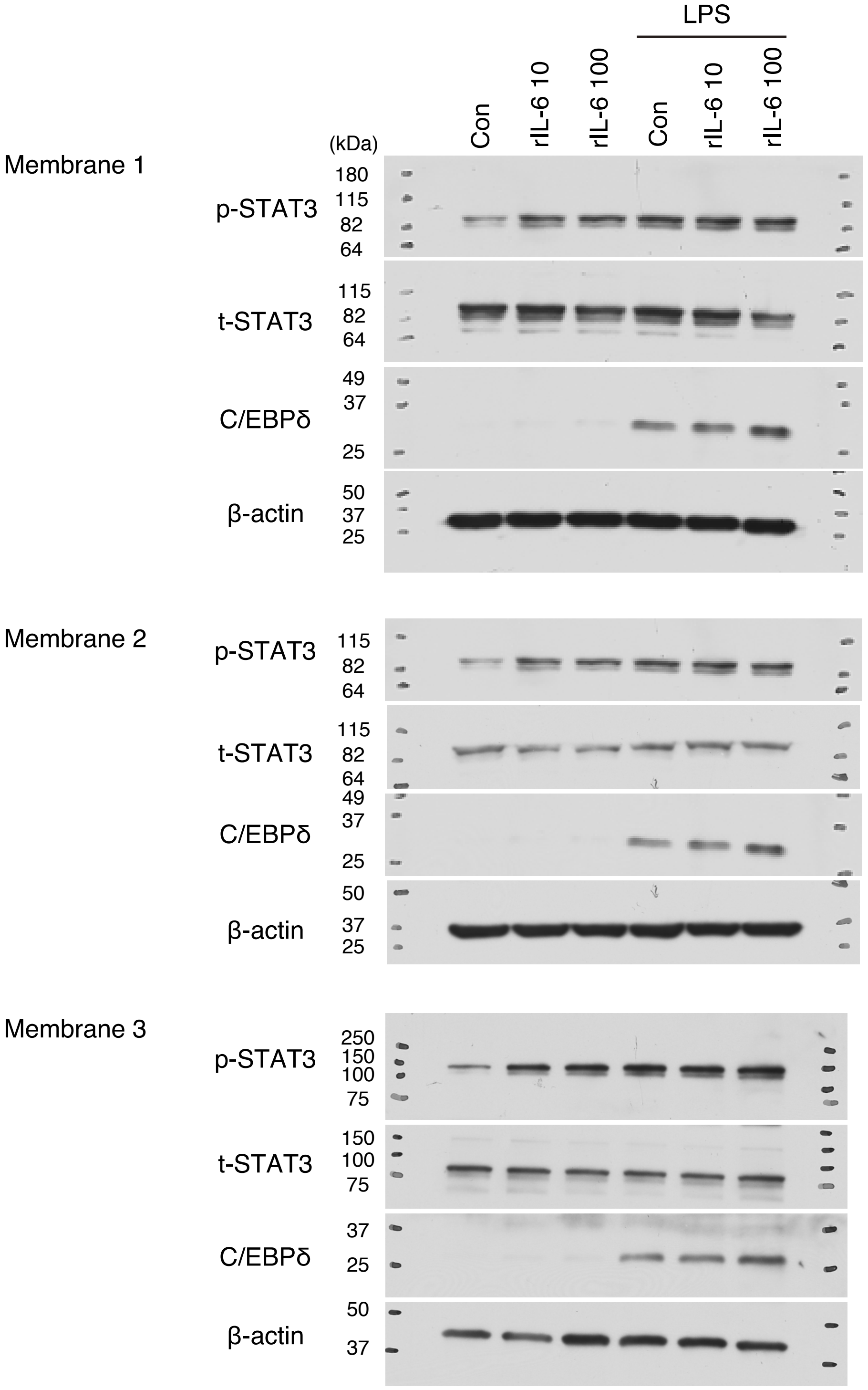

Blot images of Fig 6D

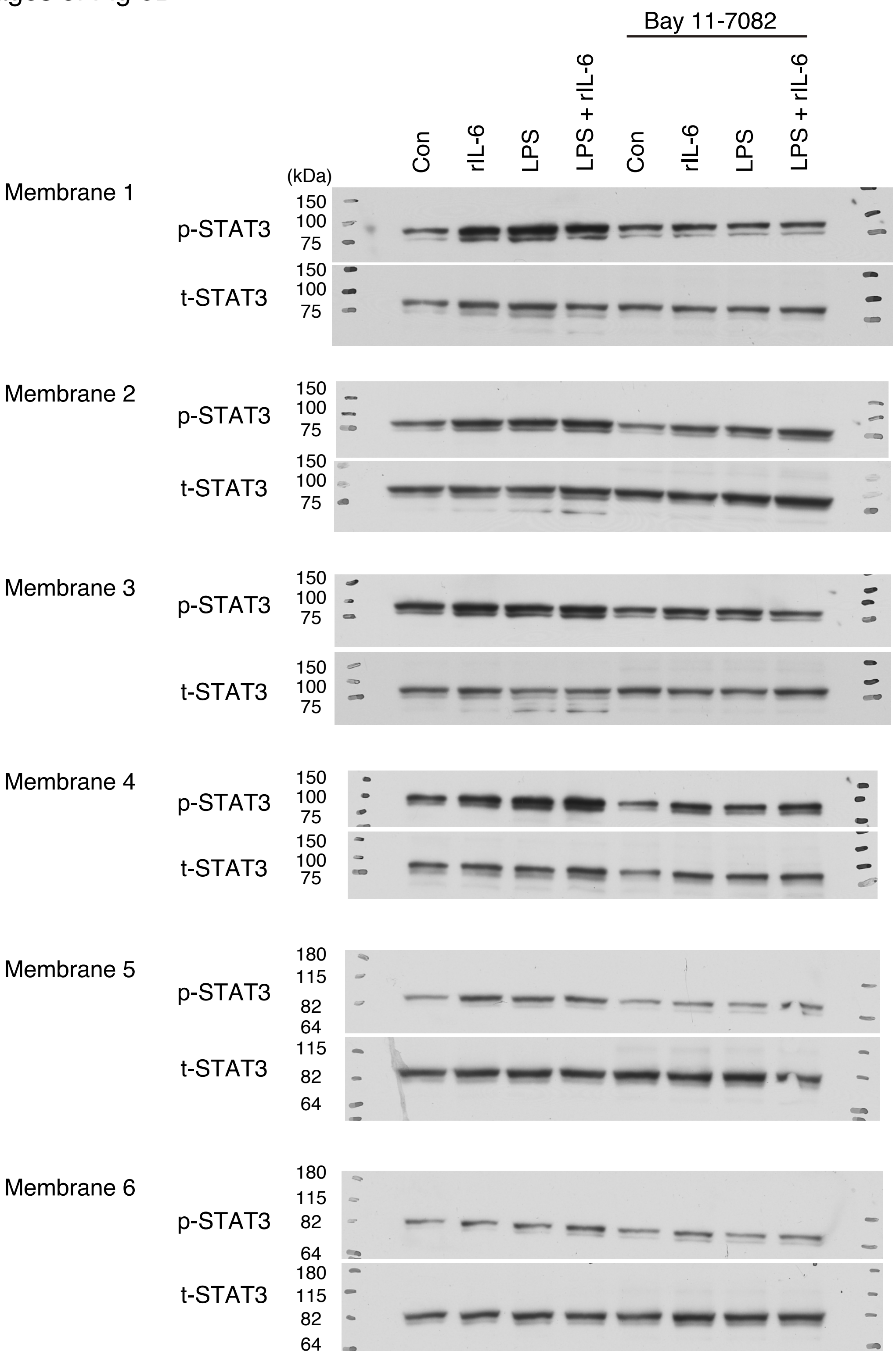

Blot images of Fig 6E

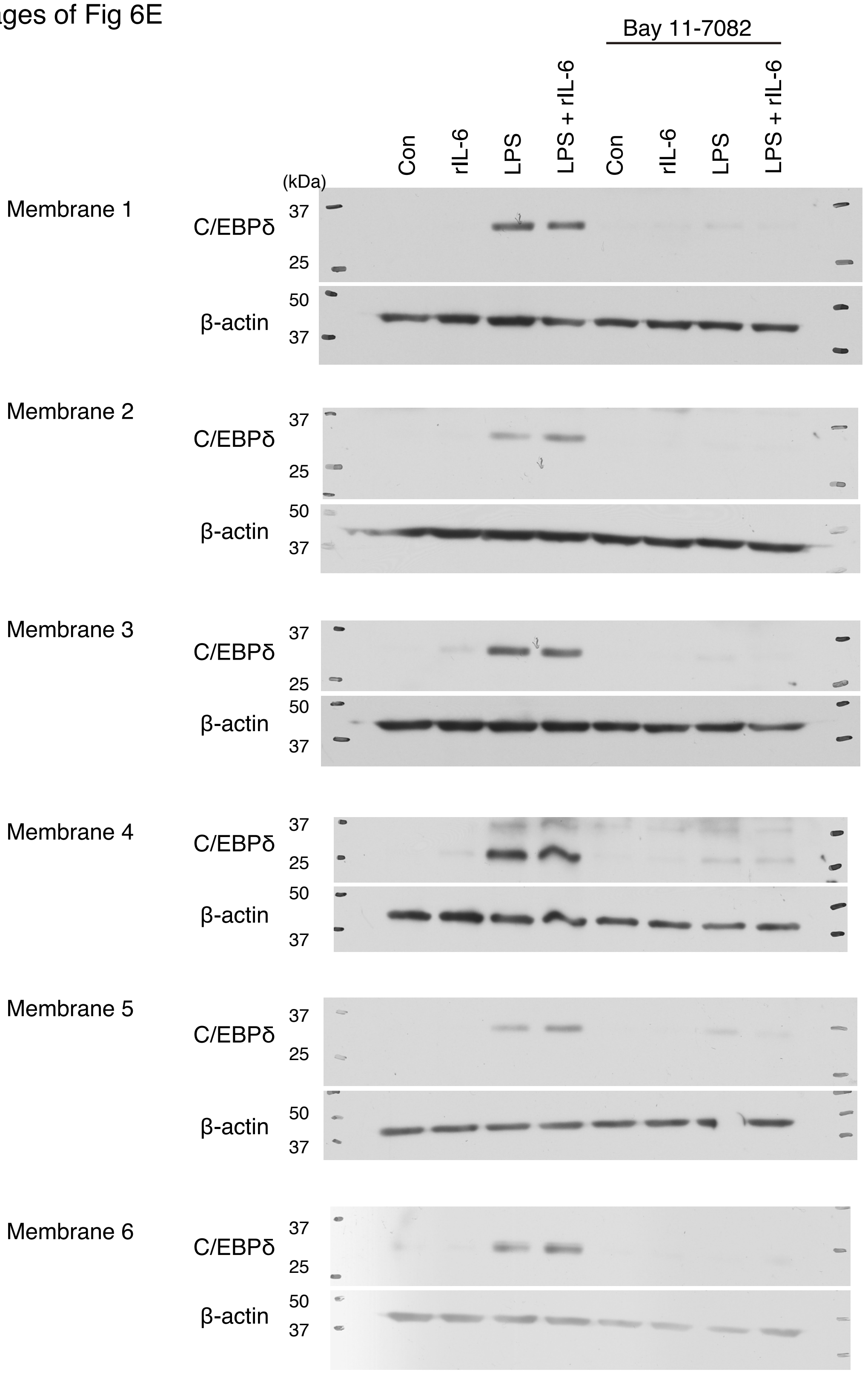

Blot images of Fig 7D

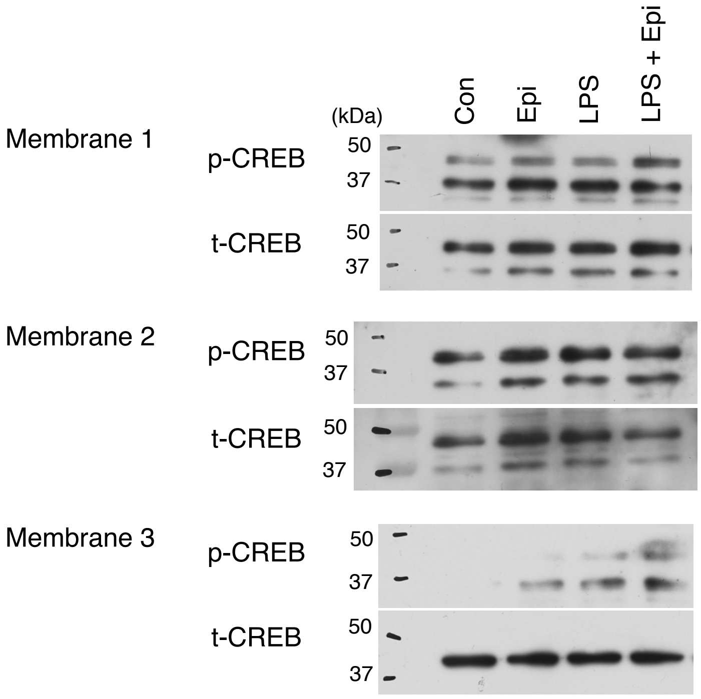

Blot images of Fig 8D

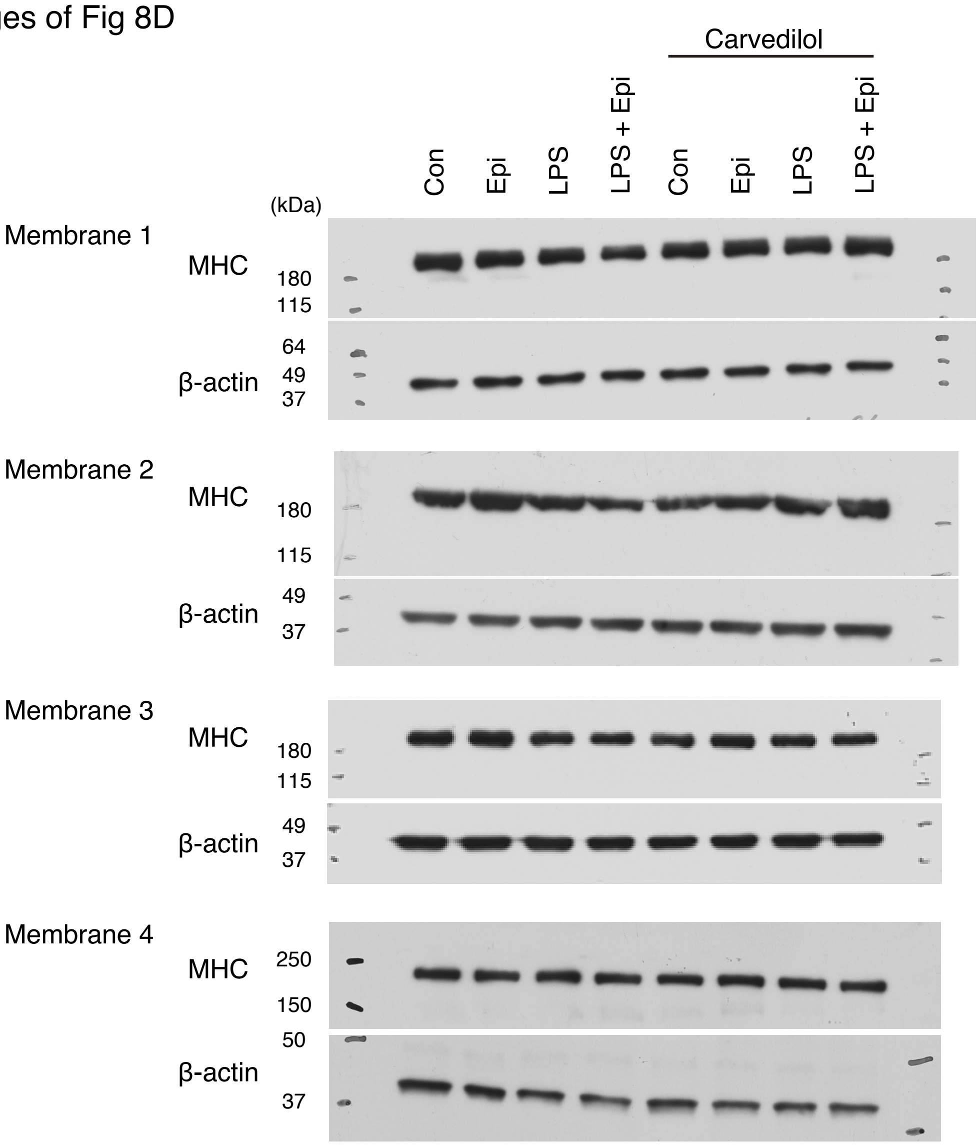

Blot images of Supplementary Figure 1A

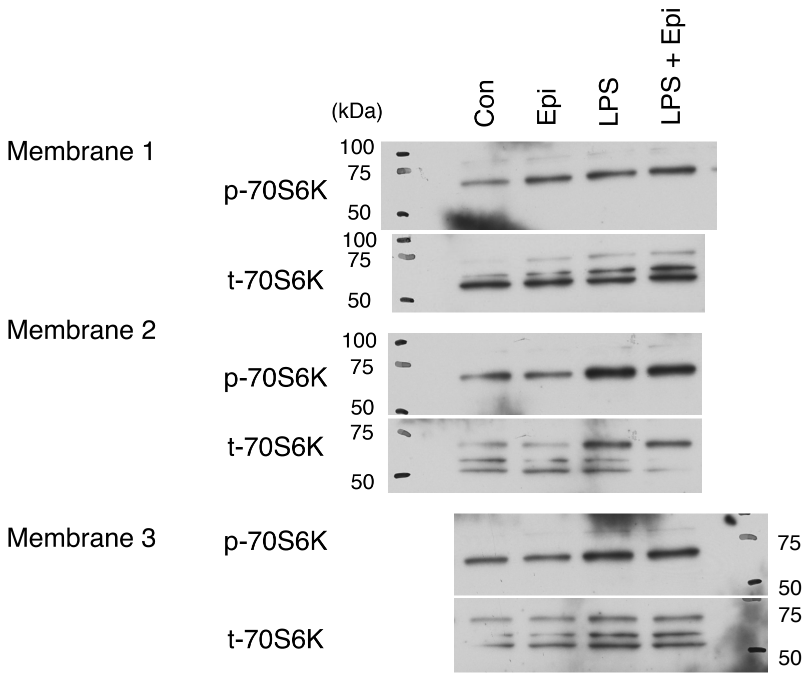

Blot images of Supplementary Figure 1B

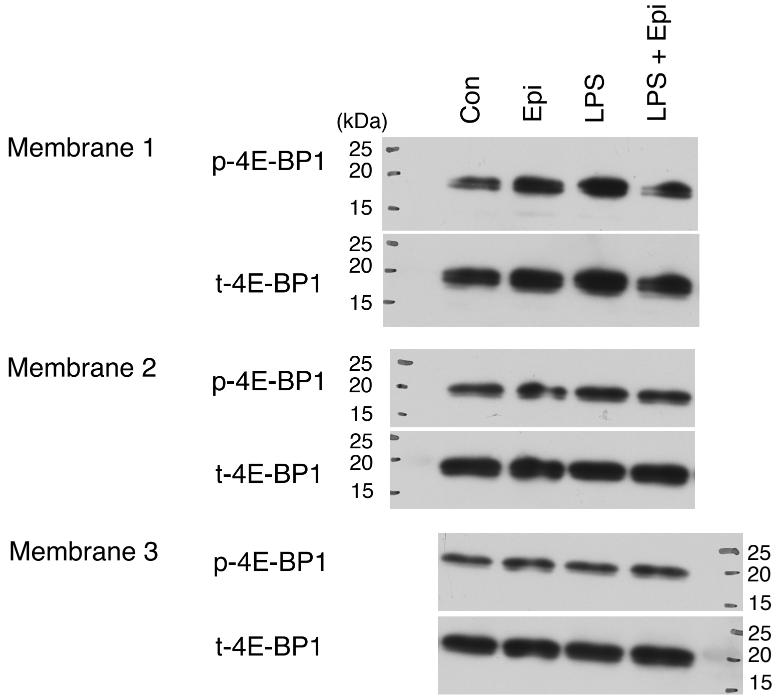

Supplement: S1 File — (PDF) [file pone.0251921.s002.pdf]
