# Supplementary material for: Activation of the β-adrenergic receptor exacerbates lipopolysaccharide-induced wasting of skeletal muscle cells by increasing interleukin-6 production
Source: PLoS One. 2021 May 18;16(5):e0251921. doi: 10.1371/journal.pone.0251921 (PMC8130926; doi:10.1371/journal.pone.0251921)
Supplement: Attachment — Submitted filename: Response to Reviewers_0419.docx [file pone.0251921.s004.docx]

April 19, 2021

Dr. Atsushi Asakura,

Editor-in-Chief,

PLOS ONE

Dear Dr. Asakura,

Thank you for inviting us to submit as revised draft of our manuscript entitled “Activation of the β-adrenergic receptor exacerbates lipopolysaccharide-induced wasting of skeletal muscle cells by increasing interleukin-6 production” to PLOS ONE. We also appreciate the time and effort you and each of the reviewers have dedicated to providing insightful feedback on ways to improve our paper. Thus, it is with great pleasure that we resubmit our article for further consideration. We have incorporated changes that reflect the detailed suggestions you have graciously provided. We also hope that our edits and the responses we provide below satisfactorily address all the issues and concerns you and the reviewers have noted. The red characters in “Revised Manuscript with Track Changes” are places of correction.

Reviewer #1: This manuscript written by Dr. Matsukawa et al. aimed to clarify the effects of catecholamines in LPS-treated myotubes, and found that the epinephrine accelerates the LPS-induced muscle wasting and their related signaling pathway. With several types of pharmacological inhibition, the authors clarified the link between the two different signaling pathways of LPS and b-AR, in relation to IL-6 and STAT signaling. These experiments were well designed, and the presented results were convincing. However, it is still unclear why the epinephrine could enhance the LPS-induced muscle wasting, while the epinephrine treatment without LPS-induction had no effect on myotubes. The reviewer raised some concerns that need to be addressed in order to strengthen the conclusions drawn by the authors.

We appreciate you taking the time to offer us your comments and insights related to the paper. We tried to improve Figure 9 to more clearly explain the predicted mechanism. We hope you find these revisions rise to your expectations.

The critical concern was that it was not clear why epinephrine could accelerate the LPS-induced muscle wasting, and could activate relevant signal genes, namely STAT3, Atrogin-1 and NF-kB. One possibility is that the number of b-AR or the binding affinity to epinephrine were increased by LPS-treatment, and those augmentation enhanced the muscle wasting. Did the authors evaluate the expressions or the sensitivity of b-AR after LPS treatment? Did LPS-induced IL-6 affected them? Clarification of these would strongly support the hypothesis.

Thank you for providing these insights. I have redrawn Figure 9 because this schematic representation might have confused you. Epinephrine activated STAT3 signaling but not NF-κB signaling, whereas LPS could activate both STAT3 and NF-κB signaling. It is assumed that STAT3 and NF-κB cooperate to promote CEBPδ and Atrogin-1 expression. We speculated that STAT3 activation by rIL-6 did not promote CEBPδ and Atrogin-1 expression in the absence of NF-κB activation by LPS. Therefore, Epinephrine could accelerate muscle wasting through the increase of STAT3 activity in the presence of LPS-induced NF-κB activation, whereas epinephrine could not induce muscle wasting in the absence of LPS stimulation.

According to your suggestion, we assessed the expression of β2-AR mRNA expression by qRT-PCR. Epinephrine and/or LPS did not significantly affect the expression of β2 -AR (The data for reviewers 1). From this result, we assumed that LPS did not affect β2 -AR expression. We administered Epi 1μM to C2C12 cells as a sufficiently high dose because Epi 10μM with LPS increased the induction of Atrogin-1 mRNA expression less than Epi 1μM with LPS. Therefore, we considered that the effect of Epi could not be explained by LPS-induced change in β2 -AR sensitivity.

In Fig. 2, the authors evaluated the proteolysis related gene expressions including Atrogin-1 and MURF, but did not describe about the protein synthesis. Since the activation of b-AR increases skeletal muscle mass as previously described, it is a question whether the treatment with both LPS and epinephrine would affect the protein synthesis.

According to your suggestion, we asessed phosphorylation of p70S6K and 4E-BP to examine whether Epinephrine and LPS could affect protein synthesis in skeletal muscle (S1 Fig). It was demonstrated that LPS and Epinephrine did not increase the phosphorylation of these proteins. Thus, we suggest that epinephrine enhances the LPS- induced muscle wasting through increase in proteolysis rather than decrease in protein synthesis (p. 14, lines 187-194).

In Fig. 5, IL-6 inhibitor suppressed LPS-induced STAT3 activation and Atrogin-1 expression. The reviewer wonders if IL-6 inhibitor could prevent the myotube atrophy in vitro along with the alteration of these signaling molecules.
In addition, since NF-kB inhibitor also decreased these expressions, I wonder if the loss of MHC protein after LPS- treatment could be attenuated by Bay11-7082, as well.

As shown in Figures 5B and 5C, the IL-6 inhibitor partially suppressed CEBPδ and Atrogin-1 expression induced LPS, whereas it abolished the effect of Epinephrine on CEBPδ and Atrogin-1 expression in the presence of LPS. These observations suggest that the IL-6 inhibitor will suppress the effect of Epinephrine on the MHC protein expression in the presence of LPS (p. 22, lines 335-339).

On the other hand, the NF-κB inhibitor abolished C/EBPδ and Atrogin-1 expression induce by LPS with or without Epi. Consistently, the data for reviewers 2 shows that the NF-κB inhibitor abolished the effect of LPS with or without Epi on the MHC protein loss.

In Fig. 6A and B, the results indicated that rIL-6 treatment (without LPS-induction) do not affect C/EBP and Atrogin-1 expression even under the situation of p-STAT3 elevation, which implies that IL-6/STAT3 signaling is independent of C/EBPd-Atrogin1 signaling. However, in Fig. 9, the authors showed that C/EBPd was the downstream of IL-6/STAT3 pathway, which was inconsistent with other results shown in the manuscript. Explanations are required for these issues. In addition, please explain why LPS+IL-6 treatment could enhance C/EBPd-Atrogin1 signaling while rIL-6 did not affect these expressions.

You have raised an important point. We agree with you and modified Figure 9 in our paper. We assume that NF-κB induces C/EBPδ and Atrogin-1 expression, whereas STAT3 does not induce their expression in itself but potentiates the effect of NF-κB. Therefore, rIL-6 did not affect C/EBPδ–Atrogin-1 signaling in the absence of NF-κB activation, whereas the NF-κB inhibitor could abolish the induction of C/EBPδ–Atrogin-1 signaling by LPS with or without Epi. Consistently with our results, it was reported that STAT3 and NF-κB cooperates to promote the expression of pro-cachectic or pro-inflammatory genes [PMID: 28264935, 29185442]. However, the precise mechanism of STAT3- NF-κB crosstalk still remains to be clarified. Further investigation about this mechanism is needed in the future (p. 23, lines 350-356).

In Fig. 6D and E, NF-kB inhibitor could abolish C/EBPd, Atrogin-1 and IL-6 expression as shown in Fig. 6 and 7, while it is still not clear whether this would affect the pSTAT3 signaling. Validation on the activation levels of STAT3 in Bay11-7082-treated myotubes is necessary.

We have added a new data showing the effect of the NF-κB inhibitor on the p-STAT3 (Figure 6D). It is demonstrated that the NF-κB inhibitor could slightly suppress LPS-induced p-STAT expression, indicating that NF-kB could partially affect p-STAT3 pathway. However, it is not clear whether NF-κB signaling affects p-STAT3 pathway directly or through increase in IL-6 production. Further investigation will be necessary to clarify the mechanism for the STAT3 activation by the NF-κB signaling (p. 17, lines 252-254).

Since epinephrine without LPS-treatment did not have any effects on myotube, it was unclear if b-AR mediated IL- 6 signaling could directly affect Atrogin-1 expression and MHC protein amount as shown in Fig. 8. To answer this question,, experiments using b-AR overexpressed C2C12 might be required.

Thank you for this suggestion. I have redrawn Figure 9 because this schematic representation might have confused you. NF-κB activation is essential to induce Atrogin-1 expression (Figure 6F). Activation of β-AR by Epi did not significantly activate NF-κB (Figure 6C). Therefore, we believe that β-AR overexpression would affect neither NF-κB activity nor Atrogin-1 expression and MHC protein amount in the absence of LPS.

Again, we appreciate all of your insightful comments. We worked hard to be responsive to them. Thank you for taking the time and energy to help us improve the paper.

Reviewer #2: This paper is an exciting manuscript by Matsukawa et al. on sepsis's hot topic and its therapeutical strategies such as catecholamines. The idea of the rationale of the experiments is correct. However, the data showed is low-quality, especially those based in western blots that should contribute to the conclusions to a high degree. Thus, this reviewer thinks that the findings are not entirely based on the manuscript results.

Specific: the western blot of the Figures 1A, 2A, 2B, 5A do not represent the changes between LPS and LPS+ Epi depicted in the graphics. Also, Figure 6D does not show the differences between LPS and LPS+IL6. Why does LPS not increase the pSTAT3 in figure 5B? Fluorescence microscopy in figure 1B presents high levels of background and complicated to see the myotubes delimitation.

We replaced some representative blots in our paper and submitted all western blots (S2 File). We believed that Figures represent the changes between LPS and LPS+Epi in the graphics. As the reviewer pointed out, Fluorescence microscopy in figure 1B presents some background. However, we consider that the delimitation of the myotubes could be adequately determined to assess their diameter. We increased the number of samples for each condition to minimize confusion.

Thank your once again for your valuable comments and suggestions. We are hopeful that our supplementary analyses and revised focus helps to improve your opinion of work.

Again, thank you for giving us the opportunity to strengthen our manuscript with your valuable comments and queries. We have worked hard to incorporate your feedback and hope that these revisions persuade you to accept our submission.

Sincerely,

SHINICHI KAI, MD.

Senior Lecturer

Department of Anesthesia, Kyoto University Hospital,

Sakyo-ku, Kyoto, 606-8507, Japan.
E-mail: [sk0627@kuhp.kyoto-u.ac.jp](mailto:sk0627@kuhp.kyoto-u.ac.jp)

Tel: +81-75-751-3433
